# The effect of harvest time of forage on carbohydrate digestion in horses quantified by in vitro and mobile bag techniques

**DOI:** 10.1093/jas/skac422

**Published:** 2022-12-28

**Authors:** Frida Lindskov Stang, Rikke Bjerregaard, Cecilia Elisabeth Müller, Åshild Ergon, Magnus Halling, Nana Wentzel Thorringer, Alemayehu Kidane, Rasmus Bovbjerg Jensen

**Affiliations:** Department of Veterinary and Animal Sciences, University of Copenhagen, DK-1870 Frederiksberg, Denmark; Department of Veterinary and Animal Sciences, University of Copenhagen, DK-1870 Frederiksberg, Denmark; Department of Animal Nutrition and Management, Swedish University of Agricultural Sciences, SE-750 07 Uppsala, Sweden; Department of Plant Sciences, Norwegian University of Life Sciences, NO-1430 Ås, Norway; Department of Crop Production Ecology, Swedish University of Agricultural Sciences, SE-750 07 Uppsala, Sweden; Department of Animal and Aquacultural Sciences, Norwegian University of Life Sciences, NO-1430 Ås, Norway; Department of Animal and Aquacultural Sciences, Norwegian University of Life Sciences, NO-1430 Ås, Norway; Department of Animal and Aquacultural Sciences, Norwegian University of Life Sciences, NO-1430 Ås, Norway

**Keywords:** equine, fructans, grass species, in situ, sugar, water-soluble carbohydrates

## Abstract

Carbohydrates in forages constitute an important part of the feed ration for all horses. The aim of the present study was to investigate the effect of harvest time on carbohydrate composition and digestion of various grass species. The experiment was divided into three parts 1) characterization of the chemical composition of experimental feeds (6 grass species: meadow fescue [MF], cocksfoot [CF], perennial ryegrass [PR], smooth bromegrass [SB], tall fescue [TF], and timothy [TI], and 3 harvest times: early, medium, and late first cut), 2) measurements of the in vitro digestion of selected experimental feeds (the 6 grass species, and 2 harvest times [early and late]) measured by in vitro gas production, and 3) in vivo digestion of selected experimental feeds (2 grass species: CF and PR, 2 harvest times [early and late]) measured by the mobile bag technique using caecum cannulated horses. An experimental field was established with plots containing each of the grass species in three replicate blocks. Grass samples were cut between 1200 and 1400 h at 4th of June (early first cut), 17th of June (medium first cut), and 1st of July (late first cut) and analyzed for crude protein (CP), neutral detergent fiber with heat stable amylase and free of residual ash (aNDFom) and water-soluble carbohydrates (WSC). The in vitro fermentation was investigated using the ANKOM RF gas production technique, where feeds were incubated for 48 h using horse caecal fluid as an inoculum. Gas production was modeled, and maximum gas production (MGP) was used to evaluate the potential digestibility of the feeds. Based on the chemical analyses and the in vitro experiment, early and late harvested CF and PR were selected for the in vivo experiment, which was conducted as a randomized 4 × 4 Latin square design including four periods, four horses and four feeds. In general, the CP content decreased whereas the aNDFom content increased as the grasses matured. The content of WSC increased in SB and TI, but decreased in CF, and fructans increased in SB, TI, PR, and TF as they matured. The in vitro MGP showed a clearer difference between harvest times than between grass species. Harvest time had larger effect on digestibility than grass species, and a high precaecal disappearance of the WSC fraction was measured by the mobile bag technique. Cocksfoot was identified as a grass species with potentially low digestibility and low WSC content and could potentially be used more for horses.

## Teaser

Grass species and harvesting time affect the nutrient content, in vitro and in vivo digestibility of forages measured with the mobile bag technique in horses. This study showed that harvesting time of forage had a larger effect on digestibility than grass species, and that fructans can potentially be digested precaecally.

## Introduction

Carbohydrates in forage grasses are divided into structural (mainly cellulose, hemicelluloses, and pectin) and nonstructural (sugars and starch). In cool-season C3-grasses, the non-structural carbohydrates (NSC) consist mainly of glucose, fructose, sucrose and fructans, together termed water-soluble carbohydrates (WSC). Grasses produce simple sugars from photosynthesis, and when produced in excess of the energy requirements of the plant for growth and development, sugars are converted to storage carbohydrates in the plant (Longland and Byrd, 2006). Fructan is the primary storage carbohydrate of cool-season C3-grasses. Fructans consist of a terminal glucose with additional fructose units linked together in branched or non-branched structures of various lengths; different grass species having different types (Pollock and Cairns, 1991).

The carbohydrates constitute a large proportion of the forage and are important energy sources for all horses. The structural carbohydrates are fermented by hindgut microbes to short-chained fatty acids (SCFA) providing the largest energy source for the horse (Janis, 1976), and the WSC are mainly digested and absorbed as glucose and fructose in the small intestine (Dyer et al., 2002; Merediz et al., 2004). Currently it is not clearly known how and where fructans are digested within the equine gastro-intestinal tract. It is assumed that fructans cannot be digested in the small intestine because horses lack the enzymes that break the linkages between the fructose molecules in fructans. Nevertheless, preliminary studies have shown that part of fructans might be digested in the stomach of the horse (Coenen et al., 2006), probably through acid hydrolysis from the acidic environment in the lower part of the stomach. Fructans have also been linked to development of laminitis in horses (Longland and Byrd, 2006), and it has also been reported that high doses (10 g/kg body weight) of nongrass fructans resulted in hindgut acidosis and laminitis in horses (Van Eps and Pollitt, 2006). Hence, more research is needed to understand the in vivo digestion of grass fructans in horses. Sugars causing a glucose and insulin response are also of interest in equine nutrition, especially for horses with insulin dysregulation (Frank et al., 2010; Lindåse et al., 2016), pituitary pars intermedia dysfunction (McGowan, 2008) or polysaccharide storage myopathy (Firshman et al., 2003; Borgia et al., 2011). These horses’ risk severe health problems with high intake of NSC and should therefore ideally be fed a diet containing less than 10% NSC of dry matter (DM), preferably lower (Borgia et al., 2011; Lindåse et al., 2018). Recommendations on how to produce forage with NSC (or WSC) concentrations below 10% of DM are currently lacking, as most of the research performed on forages and their composition and nutritive values have been focusing on ruminants where a high NSC content is desired (Humphreys et al., 2006; Shewmaker et al., 2006). Different grass species may differ in their capability to produce forages with less than 10% NSC of DM (King et al., 2012; Jensen et al., 2014) and more information is needed on how different grass species respond to different plant maturity at harvest in their NSC concentration and composition.

The structural carbohydrates and fiber digestibility should be considered when selecting grass species for forages for different types of horses. As fiber content increases digestibility decreases, and it has a major impact on the digestible energy value of the forage which decreases as the grass matures (Ragnarsson and Lindberg, 2008, 2010; Müller, 2012). Horses with high energy requirements therefore benefit from highly digestible early harvested forage (Ringmark et al., 2013), whereas horses with comparably low energy requirements benefit from a late harvested forage with a low digestibility (Harris et al., 2017). However, the grass species can affect both fiber content and digestibility despite being harvested in the same plant maturity (Särkijärvi et al., 2012). This knowledge could be used to a higher degree in equine nutrition, but more information about differences in fiber digestibility between grass species is needed, especially for harvest times that are relevant for horse requirements.

Various in vitro and in vivo techniques can be used to quantify digestion of forages in horses, each with different advantages and disadvantages. One technique commonly used is the in vitro gas production technique (IVGPT) where a positive correlation between the produced gas and digestibility has been found when using fecal contents or digesta as inoculum (Lowman et al., 1999). A disadvantage of the IVGPT is that it cannot quantify where in the gastrointestinal tract of the horse the different carbohydrate fractions are digested. As this is of interest particularly for fructans, other techniques are required. The mobile bag technique (MBT) in caecum-cannulated horses can be used to quantify the precaecal digestibility of feedstuffs and its components, even if most research using this technique has focused on starch digestion of grains (Philippeau et al., 2014; Thorringer et al., 2020) or fiber digestion (Moore-Colyer et al., 2002; Thorringer et al., 2022). It is possible to use the MBT also for digestibility studies of WSC, but as of today it is not known what the optimal pore size of mobile bags should be and how it affects the results.

The aim of the present study was therefore to quantify carbohydrate composition of various grass species harvested at different times and their digestion in horses using IVGPT and MBT, the latter with different pore sizes of bags. The hypotheses were 1) carbohydrate composition is affected by grass species and harvest time (fiber content increases and WSC decreases as the plants mature), 2) in vitro digestion is affected more by stage of maturity than grass species, 3) the in vivo digestion of glucose, fructose and sucrose but not fructans is complete in the small intestine of the horse, and 4) a mobile bag pore size of 36 µm will results in higher loss of nutrients than a pore size of 15 µm.

## Materials and Methods

### Experimental design

All housing, management, and experimental procedures followed the laws and regulations for experimental animals in Norway (i.e., Regulations on the Use of Animals in Experiments, July 2015), and the experiment was approved by the Norwegian Food Safety Authority (FOTS ID 22251). The experiment was divided into three parts 1) characterization of the chemical composition of experimental feeds (6 grass species, 3 plant maturities), 2) in vitro digestion of selected experimental feeds (6 grass species, 2 plant maturities) measured by the IVGPT, and 3) in vivo digestion of selected experimental feeds (2 grass species, 2 plant maturities) measured by the MBT using caecum cannulated horses.

### Experimental feeds

The grass species used for the experiments were grown at the Norwegian University of Life Sciences, Ås, Norway (59°39ʹ42.5″N 10°44ʹ56.0″E, 82 m above sea level, weather data is presented in Supplementary Figure S1). The species sown were meadow fescue (MF, *Festuca pratensis*, cv. Minto), cocksfoot (CF, *Dactylis glomerata*, cv. Laban), perennial ryegrass (PR, *Lolium perenne*, cv. Figgjo), smooth bromegrass (SB, *Bromus inermis*, cv. Leif), tall fescue (TF, *Festuca arundinacea*, cv. Swaj), and timothy (TI, *Phleum pratense*, cv. Grindstad). An experimental field was established with a total of 36 plots divided into three blocks, each block containing two randomly placed replicates of each grass species. The grasses were sown at the end of May 2018 with barley (*Hordeum vulgare*, cv. Salome) as a cover crop at a seed rate of 180 kg/ha and irrigated during the summer 2018. The plots were fertilized with a compound fertilizer at a rate of 80, 30, and 100 kg N ha^−1^ prior to sowing, after harvesting the cover crop, and in spring 2019, respectively. The grasses were cut with a Haldrup plot harvester at approximately 7 cm height in autumn 2018, and in 2019 the grasses were cut between 1200 and 1400 h at 4th of June (early first cut), 17th of June (medium first cut), and 1st of July (late first cut). One of the two replicate plots within a block was used for the early cut, while the other replicate plot was divided in two: one half for the middle cut and one half for the late cut. This resulted in three replicate samples of each grass species for each of the three harvest times. The biomass from each plot was mixed, and approximately 1 kg of the sample was oven-dried at 50 °C for 3 d, then milled to pass a 1-mm screen (cutting mill SM 200, Retch GmbH, Haan, Germany) and stored for later analysis.

### Animals and diets

Five caecum cannulated, 14- to 26-yr-old Norwegian Cold-blood trotter geldings with an average initial BW of 575 kg (range 545 to 631 kg) were used for the in vitro and in vivo experiments. The horses were fitted with a permanent polyvinyl chloride cannula (length ~ 15 cm; 40 mm o.d., and 30 mm i.d.) at the base of the caecum close to the ileo-caecal junction more than 10 yr before the experiment. The horses were housed in an unheated barn in 3 × 3 m individual box stalls with rubber mats and wood shavings as bedding-material. They were allowed access to a gravel paddock for approximately 6 h per day, except for days with in vivo measurements where the horses were allowed access to the paddock for approximately two hours after the measurements. The horses received 9 to 12 kg hay as-fed (timothy-meadow fescue) divided into three equal meals fed at 0600, 1400, and 1930 h (the morning meal was fed between 0700 and 0745 h on test days in the in vivo experiment). The morning meal also included 30 g/d sodium chloride and 100 g/d of a commercial mineral and vitamin supplement (Champion Multitilskudd, Felleskjøpet Fôrutvikling, Trondheim, Norway) consisting, per kg original matter, of Ca, 100 g; P, 70 g; Mg, 32 g; Cu, 840 mg; NaCl, 50 g; Zn, 2830 mg; Mn, 1530 mg; Fe, 2460 mg; I, 18 mg; Co, 6 mg; Se, 10.2 mg vitamin A, 107,000 I.U.; vitamin D, 11,300 I.U.; vitamin E, 9,600 mg; vitamin B1, 260 mg; vitamin B2, 120 mg; vitamin B6, 100 mg: vitamin B12, 0,8 mg; niacin, 270 mg; folic acid, 150 mg; biotine, 15 mg, and vitamin C, 270 mg. Water was available ad libitum from automatic water troughs in the individual box stalls, and from buckets in the gravel paddock.

#### In vitro experiment

One of the three replicates of the early and late harvested grass samples (MF, CF, PR, SB, TF, and TI) were randomly selected for the in vitro experiment. The ANKOM RF gas production system (Version 9.8.3, ANKOM Technology, Macedon, NY, USA) was used to measure the in vitro digestibility according to the recommendations from the company (ANKOM, 2022). In brief, three replicates of each of the 12 grass samples (6 grass species and 2 plant maturities), three replicates without feedstuffs (blanks), and three replicates with an internal standard (sugar beet pulp) were incubated using caecal inoculum (42 bottles in total). Approximately 1.1 g of each feedstuff was weighed into 250 mL incubation bottles. A final buffer solution was prepared by mixing 875 mL macro mineral-, 875 mL buffer-, 0.44 mL micro mineral-, and 4.4 mL resazurin-solution in addition to 1,750 mL distilled water according to Goering and Van Soest (1970) omitting the addition of trypticase. The final buffer solution was flushed with CO_2_, kept in a heated water bath at 39 °C, and 183 mL reducing solution was added to the final buffer solution. The final buffer solution was flushed with CO_2_ and a reducing solution was added, and the color changed from purple to clear, indicating that the solution was saturated with CO_2_. Each bottle with feed samples and the three blanks were filled with 66 mL of the final buffer solution and placed in a heating oven at 38 °C while caecal fluid inoculum was prepared.

Caecal inoculum was collected from three horses through their caecal cannula approximately five hours after the morning meal and stored in preheated thermos bottles for the two-minute transportation between the stable and the laboratory. The inoculum was strained through a precision woven synthetic monofilament fabric with a pore size of 200 μm (Nitex 03-200/47, SEFAR, Heiden, Switzerland) into a bottle placed in a heated water bath at 39 °C. Then 34 mL inoculum was filled into each bottle containing feed samples and final buffer solution, the headspace of the bottles was flushed with CO_2_ and then closed with the head module. The bottles were placed in an incubator (B 8420, TERMAKS, Bergen, Norway) at 39 °C on two separate shakers (Gyro rocker SSL3, STUART, Staffordshire, United Kingdom) rotating at 11 rounds per minute, and measurements started. The ANKOM RF gas production system was set to record every 10 minutes and to release gas when the pressure in the bottles exceeded 0.75 psi. After 48 h the incubation was terminated, and the pH of each bottle was measured (pH 3310, Xylem Analytics, Weilheim, Germany). The content of each bottle was poured into preweighed polyester bags (Saatifil PES 12/6, Saati S.p.A., Appiano Gentile, Italy) with a pore size of 12 μm and a surface area of approximately 200 cm^2^. During this process, the filled bags were kept in water at 5–10 °C, after which all bags were washed for 35 min using the cold-water wool-programme with no final spin in a domestic washing machine (Avantixx 7 VarioPerfect, BOSCH, Gerlingen Schillerhöhe, Germany). Bags were dried in a heating oven (TS 8430, TERMAKS, Bergen, Norway) at 45 °C for 48 h after which each bag was weighed.

#### Mobile bag experiment

The in vivo experiment was conducted as a randomized 4 × 4 Latin square design over a four-week period ­including four horses and four feed samples. The feed samples included were the early and late harvests of PR and CF. In addition to the different feed samples, two different pore sizes of the mobile bags were used (15 and 36 μm). In total, 352 mobile bags were used, with 40 replicates of each grass × harvest × pore size combination, and 4 replicates of each combination, which were used to determine the washing loss of nutrients from the bags. The size of the mobile bags was 1 × 2 × 12 cm and they were prepared as described by Thorringer et al. (2020) from a precision woven synthetic monofilament fabric with a pore size of either 15 μm (Nitex 03-15/10, SEFAR, Heiden, Switzerland) or 36 μm (Nitex 03-36/28, SEFAR, Heiden, Switzerland). A steel washer with a diameter of 10 mm was heat sealed into one end of each bag, except for bags used to determine washing loss. Each mobile bag was weighed, marked for identification, and filled with 0.5 g of feed, ensuring a feed to surface area (FSA) of 21 mg/cm^2^.

The mobile bags were intubated each week between 0700 and 0745 h before the morning meal. The bags (20 bags per horse per period) were soaked in cold tap water before they were administered into the stomach with a nasogastric tube flushed with approximately 2 L of tap water. To capture the mobile bags, a 50 cm long nylon tube containing a double-sided magnet with a diameter of 2 cm was introduced into the caecum through the cannula. The magnet was withdrawn every hour until 10 h post intubation, and bags not recovered at this time were collected in feces two to four times per day until all bags had been recovered. After collection bags were quickly rinsed with tap water and then stored at −20 °C. The mobile bags were thawed at room temperature, placed in a washing bag (35 × 28 cm, pore size approximately 1 mm), and washed for 35 min using the cold-water wool-programme with no final spin in the same domestic washing machine as described previously. Each washing bag contained mobile bags collected from the caecum of one horse (12–20 bags), and two washing bags were washed at the same time. Washing loss from mobile bags that had not been intubated was done in a similar way. Bags were then dried in a heating oven (TS 8430, TERMAKS, Bergen, Norway) at 45 °C for 48 h after which each bag was weighed. To ensure enough feed residue for chemical analysis, the mobile bags were pooled by each grass species × harvest time × pore size combination in addition to the three time-intervals where bags were found in the caecum (1–3, 4–6, and 7–10 h after bags were administered into the stomach) before further chemical analysis.

#### Chemical analysis

The experimental feeds and feed residues from the in vitro and in vivo experiments were analyzed for dry matter (DM) content by drying to a constant weight for 24 h at 103 °C (feeds) or for 48 h at 45 °C (feeds and feed residues), and samples were incinerated at 550 °C for 16 h for ash determination (feeds). The content of NDF was determined using an ANKOM200 Fibre Analyzer (ANKOM Technology, Macedon, New York, USA) using heat-stable amylase followed by combustion at 550 °C and expressed without residual ash (aNDFom). Nitrogen was determined using a Kjeltec TM 8400 (FOSS, Hillerød, Demark) and crude protein (CP) content was calculated as *N* × 6.25. Analysis of WSC, glucose, fructose, sucrose, and fructans were performed using an enzymatic-spectrophotometric method described by Udén (2006).

### Calculations and statistical analysis

#### Calculations for the in vitro experiment

The cumulative gas production measurements were converted from psi to moles by the ideal gas law: 


$$\begin{array}{l} n=p\times \frac{V}{R\cdot T} \end{array}$$

where *n* is the gas produced (moles), *p* is the pressure (kPa), *V* is the head-space volume in the bottles (L), *R* is the gas constant of 8.314472 (L ∙ kPa ∙ K^−1^ ∙ mol^−1^), and *T* is the temperature (°K). Gas produced was then converted from moles to mL by the following formula:


$$\begin{array}{l} Gas\;produced\;\left(mL\right)=n\times 22,400\;mL/mole \end{array}$$

where *n* is the gas produced (moles) and 1 mole of gas will occupy 22,400 mL at standard conditions (273.15 °K and 101.325 kPa). The gas produced in mL was then corrected for the DM content in the bottle and fitted to the monophasic model by Groot et al. (1996) using the NLIN procedure in SAS (version 9.4, SAS Institute Inc., Cary, North Carolina, USA):


$$\begin{array}{l} G=\underset{i=1}{\overset{n}{\sum }}\;\frac{A_{i}}{1+\frac{B_{i}^{C_{i}}}{t^{C_{i}}}} \end{array}$$

where *G* is the gas produced (mL/g DM), *A* is the asymptotic gas production (mL/g DM) referred to as maximum gas production (MGP), *B* is the time (h) after incubation at which half of the MGP is reached, *C* is a constant related to the shape of the curve, and *t* is the time (h) after incubation. Furthermore, the time for the maximum rate of digestion (*t*_RM_) and thereby gas production was calculated by the following formula:


$$\begin{array}{l} t_{RM}=B\times {\left(C-1\right)}^{\frac{1}{C}} \end{array}$$

where *t*_RM_ is the time (h) for maximum rate of digestion, *B* is the time (h) after incubation at which half of the MGP is reached, and C is a constant related to the shape of the associated gas production curve. The disappearance of DM after incubation was calculated as:


$$\begin{array}{l} Disappearance(\;\%\;)=\\ \frac{Feed\;\;in\;bottle\;\left(g\right)-feed\;\;residue\;after\;incubation\;(g)}{Feed\;in\;bottle\;(g)}\times 100 \end{array}$$

#### Calculations for the mobile bag experiment

The transit time of the mobile bags from stomach to recovery in the caecum was calculated using the following equation by Faichney (1975):


$$\begin{array}{l} Transit\;time=\underset{i=1}{\overset{n}{\sum }}\;t_{i}\times M_{i} \end{array}$$

where *t*_*i*_ is the time elapsed from intubation to the midpoint of the *i*th time, and i_th_ is the number of bags found at the *i*th time as a fraction of the total number of bags found. The recovery rate of mobile bags found in the caecum during the 10 h collection period was calculated as:


$$\begin{array}{l} Recovery\;rate\;(\%\;)=\frac{number\;of\;bags\;found\;in\;caecum}{total\;number\;of\;bags\;intubated}\times 100 \end{array}$$

The DM or nutrient disappearance was calculated as:


$$\begin{array}{l} Disappearance(\;\%\;)=\\ \frac{Feed\;in\;bag\;\left(g\right)-feed\;\;residue\;\;after\;collection\;of\;bag\;(g)}{Feed\;in\;bag\;(g)}\times 100 \end{array}$$

### Statistical analysis

All statistical analyses were performed using MIXED procedure in SAS (Version 9.4). Differences in chemical composition was analyzed in a model comprising the fixed effects of harvest time (early, medium, and late), grass species (SB, MF, CF, PR, TF, and TI), and their interaction. The parameters derived from the in vitro measurements (dDM, pH, A, B, C, and t_RM_) were analyzed in a model comprising the fixed effects of harvest time (early and late), grass species (SB, MF, CF, PR, TF, and TI), and their interaction. Dry matter disappearance from individual mobile bags were plotted against time, and the slope and intercept from the linear regressions were analyzed in a model comprising the fixed effects of harvest time (early and late), grass species (CF and PR), and pore size (15 and 36 µm), no interactions were tested. The disappearance of nutrients from the mobile bags were analyzed in a model comprising the fixed effects of harvest time (early and late), grass species (CF and PR), pore size (15 and 36 µm), and time (1-3, 4-6, and 7-10 h), no interactions were tested. Finally, the loss of nutrients from the mobile bags when washed was analyzed in a model comprising the fixed effects of harvest time (early and late), grass species (CF and PR), and pore size (15 and 36 µm), no interactions were tested. Horse was not included in any of the models as inoculum used in the in vitro experiment was pooled from different horses, and residue from mobile bags were pooled by each grass species × harvest time × pore size combination in addition to the three time-intervals where bags were found in the caecum (1–3, 4−6, and 7–10 h after bags were administered into the stomach). Results are presented as least square means with standard error of the mean (SEM) as a measure of variance. Effects were considered statistically different if *P* < 0.05.

## Results

### Experimental feeds

**Figure 1.**
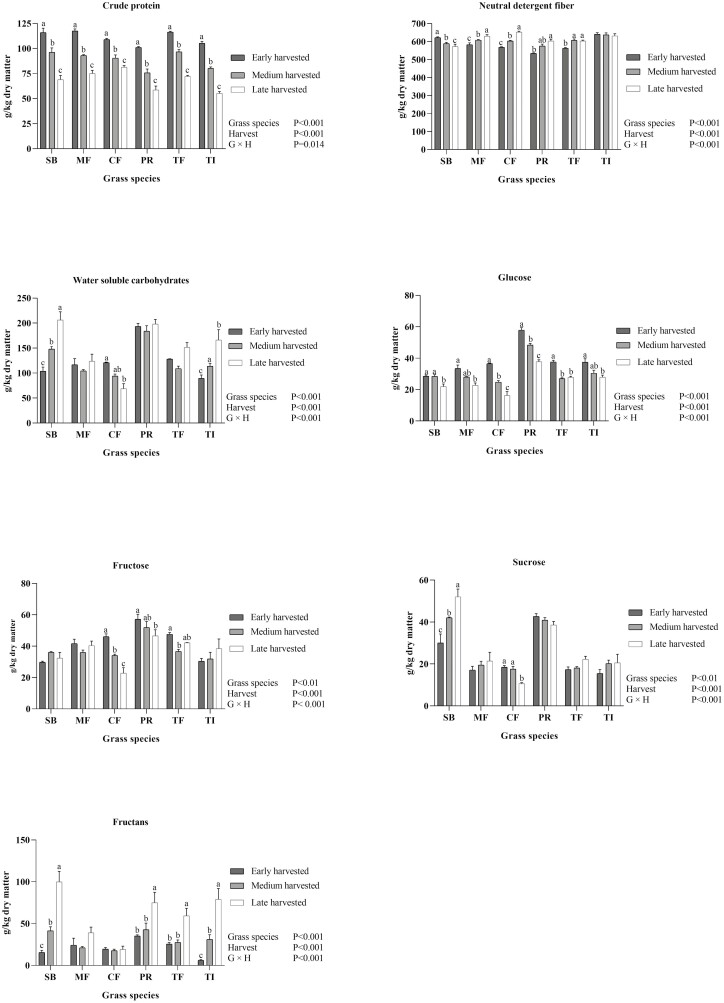
The effect of grass species (meadow fescue (MF), cocksfoot (CF), perennial ryegrass (PR), smooth bromegrass (SB), tall fescue (TF), and timothy (TI)) harvested at three different plant maturities (early, middle, and late first cut) on nutrient content. Values are presented as means ± SEM. Letters indicate statistical difference between harvest time (early, medium, and late harvest) within grass species.

The chemical composition was affected by grass species and harvest time as shown in Figure 1. Interactions between grass species and harvest time (*P* < 0.001) were present for all nutrient fractions analyzed (CP, NDF, total WSC, glucose, fructose, sucrose, and fructans). Crude protein content decreased in all grasses with increasing harvest time (Figure 1). Content of NDF increased with increasing harvesting time for all species except SB where the NDF content decreased and TI where the NDF content was similar between harvests (Figure 1). Total WSC increased for SB and TI, decreased for CF or did not change with increasing harvest time for MF, PR, and TF. In general, higher concentrations of WSC were measured for PR than for the other grass species but late harvested SB had the same WSC concentrations as PR. The lowest concentration of WSC across all harvests were present in MF and CF. Glucose concentration decreased in all grass species from early to late harvest time, and this was also the case for fructose in CF and PR. Furthermore, PR had higher levels of glucose and fructose than the other grass species. Sucrose levels were higher in SB and PR than the other grass species and in SB the level increased with increasing harvest time, while the opposite was measured for CF. Fructan levels were higher in the late harvest compared to the early and medium harvest in SB, PR, TF, and TI, while in MF and CF, the fructan levels did stay the same at all harvest times.

### In vitro experiment

The early and late harvested samples were selected for in vitro analysis based on the effect of harvest time on the chemical composition. In vitro DM digestibility parameters are presented in Table 1. The in vitro DM digestibility after 48 h of incubation was higher (*P* < 0.001) for early harvested grass than for late harvested grass. The pH after 48 h of incubation was affected by grass species (*P* < 0.001) and harvest time (*P* < 0.001), where PR had the lowest pH and CF the highest pH. Grass harvested early had lower pH after 48 h of incubation compared to late harvested grass. There was an interaction between grass species and harvest time for A (P < 0.001), B (P < 0.001), and C (P = 0.012) parameters as well as the t_RM_ (*P* < 0.001). In general, the A parameter was higher for early compared to late harvest. The A parameter was higher for MF, PR, TF, and TI than SB and CF in the early harvest, and at the late harvest SB and TF had the highest A value and CF the lowest. Parameter B was higher for late harvested SB, MF and CF, similar for TF, and lower for PR and TI compared to early harvest. In general, parameter C was highest for the early compared to the late harvest.

**Table 1.** The dry matter disappearance (dDM) and pH after 48 h of in vitro incubation and the model parameters A (the asymptotic gas production, mL/g DM), B (the time (h) after incubation at which half of A is reached), C (constant related to the shape of the curve), and t_RM_ (time for maximum rate of digestion) of six different grass species (meadow fescue (MF), cocksfoot (CF), perennial ryegrass (PR), smooth bromegrass (SB), tall fescue (TF) and timothy (TI)) harvested at two different harvesting times (early and late)

		Grass species						Harvest				P-values		
		SB	MF	CF	PR	TF	TI	Early	SEM	Late	SEM	G	H	G × H
dDM		64.4	63.8	62.3	62.2	60.8	59.8	70.1^a^	0.75	54.4^b^	0.70	0.091	<0.001	0.088
pH		6.59^bc^	6.60^b^	6.65^a^	6.56^c^	6.61^b^	6.59^bc^	6.57^b^	0.01	6.63^a^	0.01	<0.001	<0.001	NS
A	Early	164.5^b^	185.5^a^	163.1^b^	181.8^a^	178.2^a^	173.0^a^					<0.001	<0.001	<0.001
	Late	151.8^a^	140.2^bc^	112.3^d^	133.5^c^	142.0^ab^	136.9^c^							
SEM		3.8	10.2	11.5	11.0	8.7	9.1							
B	Early	9.7^ab,x^	9.0^b,x^	8.4^b,x^	8.3^b,x^	10.5^a,x^	9.6^ab,x^					<0.001	<0.001	<0.001
	Late	10.2^b,y^	12.1^a.y^	12.0^a,y^	7.6^c,y^	10.5^b,x^	9.4^b,y^							
SEM		0.34	0.71	0.89	0.21	0.43	0.27							
C	Early	1.80^a,x^	1.72^ab,x^	1.95^a,x^	1.62^b,x^	1.38^c,x^	1.79^a,x^					<0.001	<0.001	0.012
	Late	1.40^b,y^	1.44^b,y^	1.88^a,y^	1.45^b,y^	1.24^c,y^	1.35^bc,y^							
SEM		0.10	0.07	0.06	0.04	0.04	0.11							
t_RM_	Early	8.60^a,x^	7.38^b,x^	8.08^a,x^	6.17^c,x^	5.11^d,x^	8.43^a,x^					<0.001	<0.001	<0.001
	Late	5.19^c,y^	6.82^b,y^	11.02^a,y^	4.36^d,y^	3.27^e,y^	4.25^d,y^							
SEM		0.87	0.24	0.66	0.42	0.44	1.04							

Values are presented as means ± SEM. G, grass species; H, harvest time; G × H, interaction effects of grass species × harvest time.

^a,b,c,d^Values within a row are different if superscript differs (*P* < 0.05).

^x,y^Values within a column are different if superscript differs (*P* < 0.05).

### Mobile bag experiment

**Figure 2.**
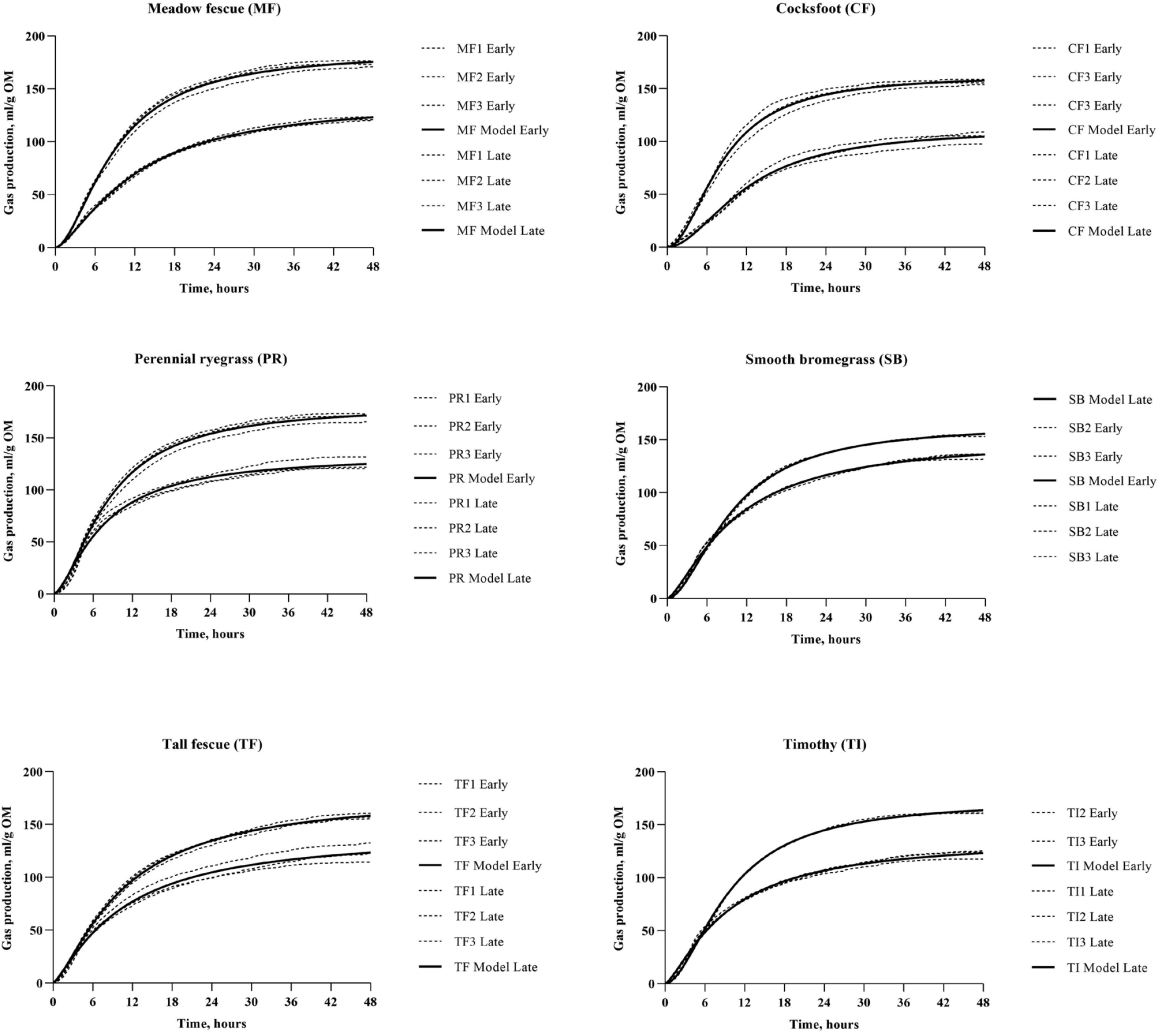
Measured (dotted lines) and modeled (solid line) in vitro gas production (ml/g organic matter (OM)) when incubating three replicates of early and late cut grasses (meadow fescue (MF), cocksfoot (CF), perennial ryegrass (PR), smooth bromegrass (SB), tall fescue (TF) and timothy (TI)) for 48 h using horse caecal fluid as inoculum. Early cut grasses had higher in vitro gas production than late cut grasses.

**Figure 3.**
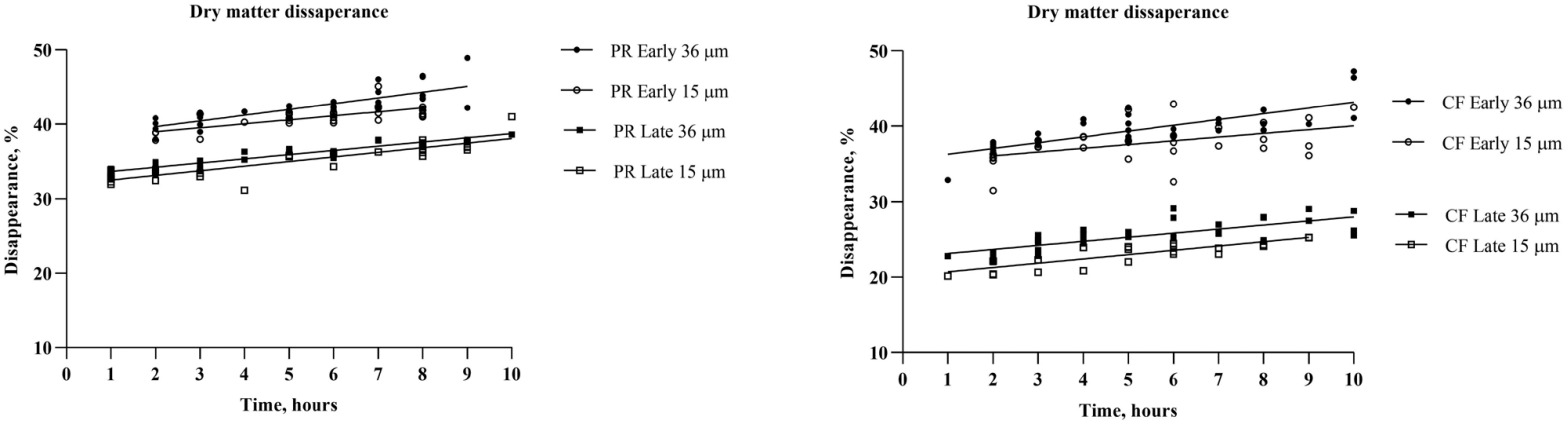
Linear regression of the dry matter disappearance from mobile bags with a pore size of 15 or 36 µm containing early and late harvested perennial ryegrass (PR) and cocksfoot (CF).

Two grass species (CF and PR) were selected for the mobile bag experiment based on their clear differences in chemical composition (Figure 1) and in vitro fermentation characteristics (Figure 2). The average transit time of the mobile bags were 4.7 ± 1.7 h, and the recovery of bags in the caecum was 78% and in total 97% of the bags were found (Supplementary Table S1). The DM disappearance from the mobile bags increased over time at the same rate (slope of lines: 0.608) but the intercept was affected by grass species and harvest time (Table 2; Figure 3) and the intercept was higher (*P* < 0.001) for the early than the late harvest time within each of the two grass species. Disappearance of DM, CP, WSC, glucose, fructose, and fructans from the mobile bags were higher (P < 0.01) for PR than for CF (Table 3). Late harvest time increased (*P* = 0.014) the disappearance of WSC and fructose compared to early harvest time. Disappearance of NDF, CP, WSC, and fructose was higher (*P* < 0.05) in bags with pore size 36 compared to 15 µm (Table 3). Nutrients were lost from the mobile bags by washing (Supplementary Table S2). Washing loss of DM, CP, WSC, glucose, fructose, and fructans was higher (*P* < 0.01) in PR than in CF. Harvest time affected (*P* < 0.05) the washing loss of aNDFom, WSC, and fructose, with the early harvest having lower loss than late harvest, despite the loss was close to 0 (Supplementary Table S2). Pore size affected (*P* < 0.05) the loss of CP, WSC, and fructose with greater loss from bags with 36 µm compared to 15 µm (Supplementary Table S2).

**Table 2.** The linear regression analysis of the relation between grass species (cocksfoot (CF) or perennial ryegrass (PR)) and harvest time (early or late) on dry matter disappearance (dDM) from mobile bags over time (X, hours).

Grass species	Harvest	Equation
PR	Early	dDM = 0.608 X + 38.01^a^
	Late	dDM = 0.608 X + 32.51^b^
CF	Early	dDM = 0.608 X + 35.29^ab^
	Late	dDM = 0.608 X + 21.37^c^

^a,b,c,d^Values within a column are different if superscript differs (*P* < 0.05).

**Table 3.** The effect of grass species (cocksfoot (CF) or perennial ryegrass (PR)), harvest time (early or late), pore size of mobile bags (15 or 36 µm), and time of recovery of mobile bags (1–3, 4–6 or 7–10 h) after administration on nutrient disappearance (%) from mobile bags

	Grass species				Harvest time				Pore size				Time						*P*-values			
	CF	SEM	PR	SEM	Early	SEM	Late	SEM	15	SEM	36	SEM	1-3	SEM	4-6	SEM	7-10	SEM	G	H	P	T
DM	31.3	2.2	38.3	1.0	39.8	0.7	29.8	1.8	33.9	2.0	35.7	2.0	33.0	2.5	35.0	2.4	36.5	2.5	<0.001	<0.001	0.098	0.036
aNDFom	7.0	1.0	6.4	0.7	8.8	0.8	4.5	0.4	5.7	0.8	7.7	0.9	5.1	0.7	6.5	1.1	8.4	1.1	0.29	<0.001	<0.001	<0.001
CP	71.6	1.3	77.0	1.7	78.1	1.3	70.4	1.3	73.2	1.7	75.2	1.7	70.0	1.9	75.8	2.1	77.0	1.6	<0.001	<0.001	<0.001	<0.001
WSC	98.5	0.3	99.2	0.2	99.1	0.2	98.6	0.3	98.7	0.4	99.1	0.2	97.8^b^	0.4	99.2^a^	0.1	99.7^a^	0.2	<0.01	0.028	0.056	<0.001
Glucose	99.0	0.4	99.0	0.2	98.7	0.3	99.4	0.2	98.9	0.3	99.2	0.3	98.6	0.6	99.0	0.2	99.5	0.3	0.98	0.14	0.41	0.24
Fructose	99.1	0.2	99.4	0.2	99.6	0.1	98.9	0.2	99.2	0.2	99.2	0.2	98.7	0.2	99.4	0.1	99.6	0.2	0.062	<0.001	0.96	<0.001
Sucrose	93.2	1.8	99.2	0.4	98.1	0.8	94.2	1.9	95.5	1.8	96.9	1.4	93.6	2.7	97.8	0.8	97.2	1.7	<0.01	0.025	0.37	0.098
Fructan	99.4	0.4	98.7	0.3	99.0	0.4	99.1	0.4	99.1	0.3	99.0	0.4	98.2	0.6	99.5	0.2	99.5	0.2	0.12	0.76	0.76	0.027

Values are presented as means ± SEM. G, grass species; H, harvest time; P, pore size; T, time of recovery of mobile bags.

DM, dry matter; CP, crude protein; WSC, water-soluble carbohydrates; aNDFom, neutral detergent.

Fiber assayed with heat-stable amylase and expressed without residual ash.

## Discussion

This study aimed to quantify carbohydrate composition and digestion of various grass species harvested at different times. It was demonstrated that harvesting time affected chemical composition of grasses and their digestibility in different ways, but also that different grass species harvested at the same date had different digestibility. A later harvest time of the different grass species clearly reduced the digestibility as shown using the IVGPT. Furthermore, the precaecal digestibility of the WSC fraction of PR and CF was high when quantified using the MBT. The methodological possibilities and limitations as well as practical application of the results are discussed in the following.

### Experimental feeds

The CP content decreased in all grass species and the aNDFom content increased in MF, CF, PR, and TF with later harvesting, which is in accordance with results from other studies performed in comparable climates (Ragnarsson and Lindberg, 2008, 2010; Müller, 2012). In the late harvested SB, the grass had lodged, making it difficult to harvest the lower fiber rich parts of the grass and this may explain the decrease in aNDFom from early to late harvest time (Griggs et al., 2007). The content of WSC, especially glucose and sucrose, in grass is of relevance for horses with insulin dysregulation (Frank et al., 2010; Lindåse et al., 2016), pituitary pars intermedia dysfunction (McGowan, 2008), or polysaccharide storage myopathy (Firshman et al., 2003; Borgia et al., 2011). Furthermore, experimentally induced hindgut acidosis using large amounts of fructans has resulted in development of laminitis (Van Eps and Pollitt, 2006), although the fructan used in that study was not of grass origin. In general, WSC was higher for PR than the other grass species, and fructans increased in SB, PR, TF and TI at the late harvest but not in MF and CF. In a more southern location (Utah, USA), Jensen et al. (2014) found PR to have high WSC, but they also found an increase of fructans in CF at later harvests. Differences between the studies may be due to differences in e.g., temperature, photoperiod, N availability, and cultivar. Variations in content of fructans have been found between cultivars of CF adapted to different climates (Sanada et al., 2007), and it would be relevant to test different cultivars of the different grass species. In contrast to PR, CF, and MF might therefore be suitable for horses prone to the abovementioned health conditions because of the relatively low content of WSC, and it should be investigated further. In all grass species except PR, an early harvest was also a possibility to reduce levels of WSC and fructans. This will however also result in a higher overall digestibility of the grass and a high energy value, which is undesired for horses with comparably low energy requirements as it reduces the amount of forage needed to cover energy requirements which in turn decrease forage intake time (Müller, 2011). Furthermore, it has been found that horses may find CF less palatable compared to other grass species when grazing (Allen et al., 2013) and this could potentially also be used to reduce feed intake in horses.

### The in vitro experiment

The in vitro measurements reflect the total tract digestibility, and in combination with the chemical composition of the grass samples, the in vitro experiment was used for selection of grass samples to be used for the mobile bag experiment. The DM disappearance in the in vitro experiment decreased from early to late harvest (70.1% vs. 54.4%) which is in accordance with results from in vivo studies where digestibility of grass forages in horses decreased with increasing plant maturity at harvest (Ragnarsson and Lindberg, 2008, 2010; Müller, 2012). No difference in DM disappearance was found between grass species within harvest time, but the MGP indicated differences between the grass species. Early and late harvested CF had the lowest MGP indicating a generally low digestibility of CF. The MGP differed more between late than early harvested grass samples, and SB had the highest MGP at the late harvest probably due to a high content of WSC. Differences in nutrient content results in different in vitro fermentations and model parameters (A, B, C, and t_RM_) differed between grasses. However, not all these parameters can be transferred directly to the gastrointestinal physiology of the horse. Here the feed undergoes enzymatic digestion in the stomach and small intestine before residual nutrients not absorbed in the small intestine are potentially fermented in the hindgut. Hence, a predigestion step before the in vitro fermentation would be required. This has not been standardized for horses, but it should be considered in future studies. A predigestion step would also be useful for quantifying the in vitro prececal digestion of feedstuffs like the method suggested by Tilley and Terry (1963). Still, MGP and DM disappearance give an indication of the potential total tract digestibility of the grass species. The pH values measured after 48 h of in vitro incubation cannot be extrapolated directly to in vivo hindgut pH values as the buffer solution will buffer the in vitro system. However, a higher DM disappearance with a potentially higher production of SCFA was reflected in a lower pH. The traditional digestibility studies with total collection of feces gives a more accurate estimate of the apparent total tract digestibility, but it does not distinguish where in the gastrointestinal tract digestion takes place, similar to the IVGPT. In this study, the in vitro measurements were used successfully to screen a large number of grass samples, as not all could be tested in vivo using the MBT. Based on the chemical composition and in vitro measurements, CF and PR were selected as highly contrasting grasses with distinct differences in WSC content and DM disappearance.

### The mobile bag experiment

The disappearance of DM, CP, and aNDFom decreased from early to late harvest in accordance with results from other studies where digestibility decreased with increasing plant maturity (Ragnarsson and Lindberg, 2008, 2010; Müller, 2012; Särkijärvi et al., 2012). The effect of harvest time on dDM was more pronounced for CF than PR, which might be explained by a faster morphological development resulting in a greater increase in aNDFom across harvest times for CF compared to PR. It was found that the precaecal disappearance of aNDFom was low, as the precaecal fermentation has been described as limited (Moore-Colyer et al., 2002; Varloud et al., 2004; Brøkner et al., 2012). The precaecal disappearances of glucose, sucrose and fructose were all above 93% which was expected, but there are no published studies (to the knowledge of the authors) where MBT has been used to study the digestion of WSC in horses previously. The high precaecal disappearance of fructans (>98%) was however not expected. Nutrients disappearing from the mobile bags might not be digested and absorbed in the small intestine, but these results indicate that grass fructans are easily available for degradation precaecally, and it is probable that microbes in the stomach and/or small intestine ferment fructans in these compartments. Additionally, a relatively large proportion (more than ~70%) of the fructans disappeared from the mobile bags by washing them (Supplementary Table S2), confirming that fructans are easily available for degradation precaecally. A preliminary study has shown that a part of fructans is digested in the stomach of the horse (Coenen et al. 2006), probably through acid hydrolysis from the acid environment in the lower part of the stomach. Results from in vitro studies (Ince et al., 2014; Strauch et al., 2017) have also indicated that fructans might be degraded precaecally by acidic hydrolysis and/or fermentation. Knowledge on which of the WSC fractions that gives the lowest responses on blood glucose and insulin levels would be relevant to investigate in future studies in relation to horses with e.g., insulin dysregulation.

Mobile bags were recovered at different time points after administration in the current study. This is of importance as a longer time within the gastrointestinal tract can potentially increase the disappearance of nutrients (Hymøller et al., 2012; Thorringer et al., 2020). Mobile bags were therefore pooled according to time of recovery and not horses in this study, as suggested by Thorringer et al. (2020). A linear relationship between disappearance of DM and time to recovery of bags was found, however, the slope of the curves did not differ, only the intercept. Early harvested grass had a greater ­intercept than late harvested grass due to higher content of soluble nutrients such as WSC and CP. This effect of time should be addressed in studies using the MBT as recovery time of bags might vary (could be affected by e.g., diet composition and individual horse). Another methodological aspect of the mobile bag technique is the pore size of the bags. The technique is not standardized, and different pore sizes and FSA (mg feed/cm^2^) have been used previously (Moore-Colyer et al., 2002; Thorringer et al., 2020). The Nordic feed evaluation system for ruminants (Åkerlind et al., 2011) recommends a pore size of 15 μm and a FSA of 5–7 and 10–15 mg feed/cm^2^ for roughages and concentrates, respectively. Thorringer and Jensen (2021) recommends not to use a FSA of more than 20 mg/cm^2^, to still have enough residue for analyses and not compromising the disappearance of nutrients from the bags. In this study, pore size influenced some nutrients, and it is recommended to use a pore size of 15 μm rather than 36 μm in future studies investigating the precaecal digestion of grass and other fibrous feedstuffs.

## Conclusion

This study demonstrated that both grass species and harvesting time affect the chemical composition of the grasses. In general, the CP decreased and aNDFom increased as the grasses matured. The content of WSC increased in SB and TI, but decreased in CF, and fructans increased in SB, TI, PR, and TF as they matured. The in vitro technique used showed clearer differences between harvesting time than between grass species. This technique can be used to screen a large number of samples within a short time. However, the in vitro fermentation does not reflect the gastrointestinal physiology of horses and a predigestion step should be considered in future studies. The mobile bag technique showed a high precaecal disappearance of the WSC fraction in grasses, including fructans. However, nutrients disappearing from the mobile bags are not necessarily digested precaecally, but the results show that fructans might be accessible for microbial fermentation and/or acid hydrolysis before reaching the hindgut. Harvesting time had larger effect than grass species on digestibility, but different grass species also influenced digestibility and composition. More specifically, CF consistently had lower and PR higher digestibility and WSC content. Further studies on grass species suitable for different horse categories should include the differences between grass species and focus on how plant maturity at harvest can be used to achieve the desired nutrient content and digestibility in the forage.

## Supplementary Data

Supplementary data are available at *Journal of Animal Science* online.

Supplementary Figure S1. Weather data showing daily average, minimum (Min.) and maximum (Max.) temperature (temp.) in ^°^C as well as daily rain in mm during the experiment. Vertical lines indicate days for harvesting early, medium, and late first cut samples. Historical weather data is obtained from an online weather service (www.yr.no).

skac422_suppl_Supplementary_Figure_S1

skac422_suppl_Supplementary_Tables

skac422_suppl_Supplementary_Figure_Legend

## Abbreviations

A: asymptotic gas production (mL/g DM)
B: time after incubation at which half of the maximum gas production is reached
C: constant related to the shape of the curve
CF: cocksfoot
CP: crude protein
G: gas produced (mL/g DM)
MGP: maximum gas production
MF: meadow fescue
*Mi*: bags found at the ith time as a fraction of the total number of bags found
NDF: neutral detergent fibre
aNDFom: NDF assayed with heat-stable amylase and expressed without residual ash
NSC: Non-structural carbohydrates
PR: perennial ryegrass
SB: smooth bromegrass
TF: tall fescue
t: time (h)
t_RM_: time elapsed from intubation to the midpoint of the ith time
TI: timothy
t_RM_: time (h) for maximum rate of digestion
WSC: water-soluble carbohydrates
