# Supplementary figures and images for: The effect of harvest time of forage on carbohydrate digestion in horses quantified by in vitro and mobile bag techniques
Source: J Anim Sci. 2022 Dec 28;101:skac422. doi: 10.1093/jas/skac422 (PMC9904184; doi:10.1093/jas/skac422)

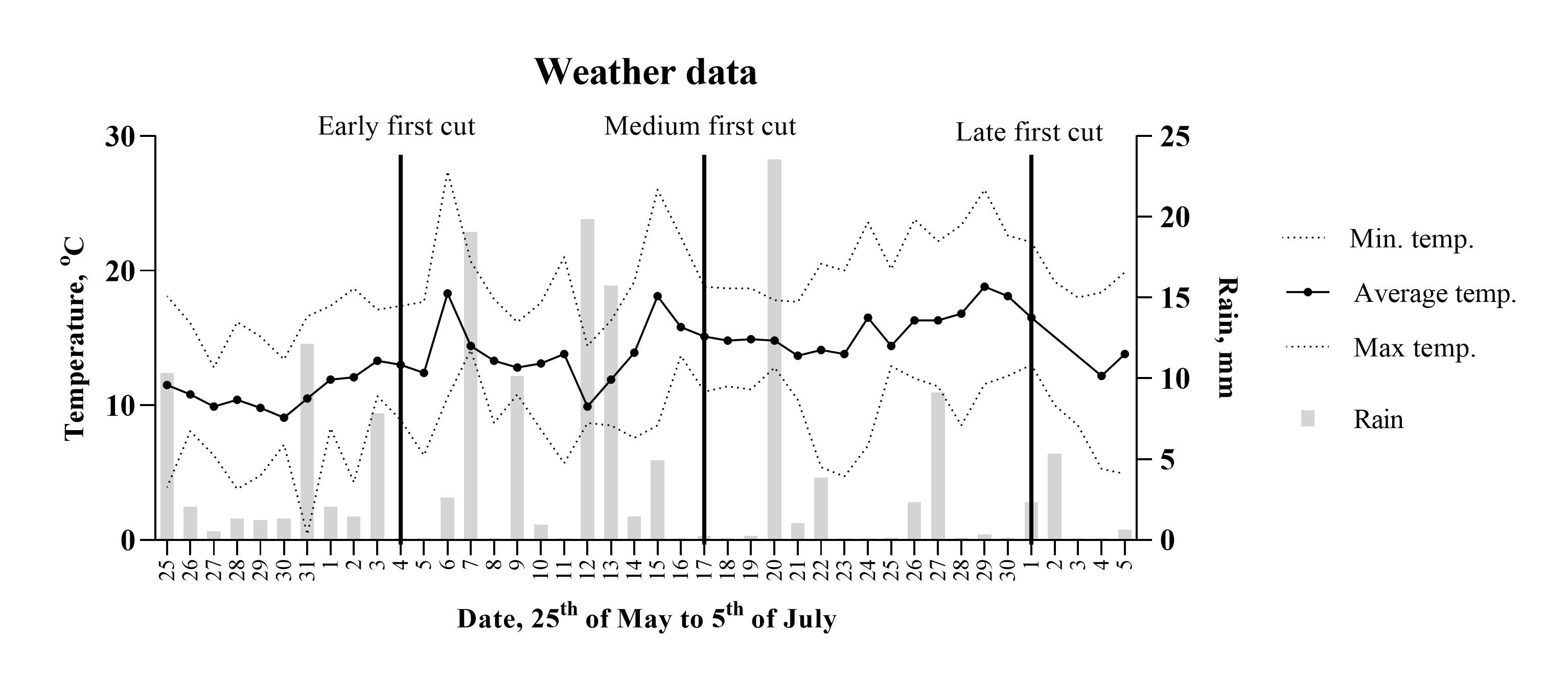

Supplement: skac422_suppl_Supplementary_Figure_S1 [file skac422_suppl_supplementary_figure_s1.jpeg]
