# Supplementary material for: The effect of harvest time of forage on carbohydrate digestion in horses quantified by in vitro and mobile bag techniques
Source: J Anim Sci. 2022 Dec 28;101:skac422. doi: 10.1093/jas/skac422 (PMC9904184; doi:10.1093/jas/skac422)
Supplement: skac422_suppl_Supplementary_Tables [file skac422_suppl_supplementary_tables.docx]

Supplementary Table S1. The number of bags recovered in faeces, precaecal (including time interval of recovery, 1-3, 4-6 or 7-10 h) and in total, when administering bags with early or late harvested cocksfoot (CF) or perennial ryegrass (PR) in mobile bags with a pore size of either 15 or 36 µm

| Grass species | CF |  |  |  | PR |  |  |  |
| --- | --- | --- | --- | --- | --- | --- | --- | --- |
| Harvest | Early |  | Late |  | Early |  | Late |  |
| Pore size, µm | 15 | 36 | 15 | 36 | 15 | 36 | 15 | 36 |
| 1-3 h, n | 10 | 11 | 7 | 12 | 5 | 12 | 13 | 14 |
| 4-6 h, n | 12 | 11 | 12 | 11 | 11 | 10 | 5 | 8 |
| 7-10 h, n | 9 | 11 | 7 | 14 | 12 | 11 | 12 | 6 |
| Pre-caecal, n (%) | 31 (78) | 33 (83) | 28 (70) | 37 (93) | 28 (70) | 33 (83) | 30 (75) | 28 (70) |
| Faeces, n | 8 | 6 | 10 | 3 | 10 | 6 | 7 | 12 |
| Total, n (%) | 39 (98) | 37 (93) | 38 (95) | 40 (100) | 38 (95) | 39 (98) | 37 (93) | 40 (100) |

Supplementary Table S2. The effect of grass species (cocksfoot (CF) or perennial ryegrass (PR)), harvest time (early or late), and pore size of mobile bags (15 or 36 µm) after administration on nutrient disappearance (%) from mobile bags after only washing. Values are presented as means ± SEM. G, grass species; H, plant maturity at harvest; P, pore size

|  | Grass species | | | | Harvest time | | | | Pore size | | | | P-values | | |
| --- | --- | --- | --- | --- | --- | --- | --- | --- | --- | --- | --- | --- | --- | --- | --- |
|  | CF | SEM | PR | SEM | Early | SEM | Late | SEM | 15 | SEM | 36 | SEM | G | H | P |
| DM | 15.9 | 2.4 | 26.5 | 0.8 | 23.3 | 2.3 | 19.1 | 4.1 | 19.8 | 3.5 | 22.6 | 3.4 | <0.01 | 0.89 | 0.25 |
| aNDFom | -0.45 | 0.33 | 0.06 | 0.7 | -0.8 | 0.5 | 0.5 | 0.4 | -0.75 | 0.52 | 0.36 | 0.41 | 0.28 | 0.051 | 0.031 |
| CP | 24.8 | 1.4 | 32.4 | 1.3 | 28.2 | 2.5 | 29.0 | 2.6 | 26.6 | 2.4 | 30.6 | 2.2 | <0.01 | 0.48 | 0.023 |
| WSC | 69.7 | 3.4 | 88.0 | 3.1 | 75.5 | 4.8 | 82.1 | 6.9 | 75.2 | 6.9 | 82.5 | 4.6 | <0.001 | 0.014 | 0.023 |
| Glucose | 68.6 | 3.7 | 86.2 | 2.9 | 73.3 | 5.3 | 81.5 | 6.0 | 73.8 | 6.6 | 81.1 | 4.7 | <0.01 | 0.082 | 0.062 |
| Fructose | 95.1 | 5.6 | 96.0 | 1.5 | 90.3 | 3.5 | 100 | 1.8 | 94.3 | 5.0 | 96.9 | 2.8 | <0.001 | 0.014 | 0.020 |
| Sucrose | 70.3 | 2.5 | 87.4 | 2.2 | 78.6 | 3.3 | 79.1 | 7.0 | 76.6 | 5.7 | 81.1 | 4.9 | 0.85 | 0.080 | 0.60 |
| Fructan | 73.9 | 3.1 | 89.0 | 2.2 | 78.1 | 4.6 | 84.8 | 4.9 | 78.6 | 5.6 | 84.3 | 4.0 | <0.01 | 0.89 | 0.25 |

DM, dry matter; CP, crude protein; WSC, water soluble carbohydrates; aNDFom, neutral detergent

fibre assayed with heat-stable amylase and expressed without residual ash
