# Supplementary material for: The effect of harvest time of forage on carbohydrate digestion in horses quantified by in vitro and mobile bag techniques
Source: J Anim Sci. 2022 Dec 28;101:skac422. doi: 10.1093/jas/skac422 (PMC9904184; doi:10.1093/jas/skac422)
Supplement: skac422_suppl_Supplementary_Figure_Legend [file skac422_suppl_supplementary_figure_legend.docx]

**Figure legends – Supplementary Figure S1**

Supplementary Figure S1. Weather data showing daily average, minimum (Min.) and maximum (Max.) temperature (temp.) in ^○^C as well as daily rain in mm during the experiment. Vertical lines indicate days for harvesting early, medium, and late first cut samples. Historical weather data is obtained from an online weather service (www.yr.no)
